# A novel ultrasound-responsive cluster bomb system for efficient siRNA delivery in brain^☆^

**DOI:** 10.1016/j.ultsonch.2025.107446

**Published:** 2025-06-25

**Authors:** Tianyu Guo, Feihong Dong, Jingyi Yin, Xinnan Wang, Pengting Min, Jiabin Zhang, Heping Cheng, Jue Zhang

**Affiliations:** a State Key Laboratory of Membrane Biology, National Biomedical Imaging Center, Peking-Tsinghua Center for Life Sciences, Institute of Molecular Medicine, College of Future Technology, Peking University, Beijing 100871, China; b Academy for Advanced Interdisciplinary Studies, Peking University, Beijing 100871, China; c College of Engineering, Peking University, Beijing 100871, China; d National Biomedical Imaging Center, Peking University, Beijing 100871, China; e Research Unit of Mitochondria in Brain Diseases, Chinese Academy of Medical Sciences, PKU-Nanjing Institute of Translational Medicine, Nanjing 211899, China

**Keywords:** Gene drug, Nanoparticles, Nanodroplets, Blood-brain barrier, Ultrasound

## Abstract

RNA-based therapeutics using RNA interference have become a research hotspot for brain tumors and neurodegenerative diseases with the advancement of nanocarrier delivery technology. However, even with specific modifications, RNA-loaded nanoparticles face significant challenges in effectively crossing the blood-brain barrier (BBB) to achieve precise delivery of therapeutic agents to the brain. Focused ultrasound combined with microbubbles and nanodroplets has emerged as a promising approach for temporarily opening the BBB. However, the low drug loading capacity and fixed stimulation focus of these methods limit their integration with current nano-drug delivery systems. Herein, we introduced a fluorinated surfactant and developed an ultrasound-responsive siRNA delivery carrier that contains nanodroplets loaded with siRNA-carrying nanoparticles (siRNA@NP@ND), termed as “ultrasound-responsive ’cluster bomb’ nanoplatform”. Under precise and flexible guidance and stimulation through a programmable diagnostic ultrasound, siRNA@NP@ND demonstrated over a seventy-fold increase in efficiency for delivering siRNA to the mouse brain. Additionally, Evans blue staining and hematological analysis indicated that ultrasound-triggered cavitation could reversibly open the BBB for up to 48 h without causing significant immune or inflammatory responses. The minor intracranial hemorrhage resulting from this process was also shown to be recoverable. Our research provides an advanced and controllable delivery platform for gene therapy of intracranial central nervous system diseases.

## 1 Introduction

Intracranial diseases, including brain tumors and neurodegenerative diseases, pose a critical challenge to global healthcare [1]. Especially malignant gliomas (e.g., glioblastoma multiforme, GBM), are among the most lethal types of intracranial diseases [2]. Although surgical resection, radiation therapy, and chemotherapy are the primary treatments for brain tumors, the diffuse infiltrative nature of brain tumors and the resistance of tumor cells to radiotherapy and chemotherapy result in extremely poor prognosis for patients, with an average survival time of only 12 to 18 months [3]. In recent years, with the rapid development of molecular biology and gene therapy technologies, RNA interference (RNAi) technology has emerged as an innovative therapeutic approach and has garnered widespread attention [4,5]. Compared to conventional drug treatments, RNAi therapy enables precise regulation of gene expression at the mRNA or protein level, offering significant advantages such as high efficiency, strong specificity, and minimal side effects [[6], [7], [8]]. It has shown immense potential, particularly in the treatment of genetic diseases, cancer, viral infections, and neurodegenerative diseases [[9], [10], [11]]. However, naked siRNA is easily degraded in blood and struggles to penetrate cell membranes to reach target cells [12].

Currently, nanocarrier-based drug delivery systems represent a primary strategy to address the challenges of siRNA delivery. Cationic nanocarriers protect siRNA from degradation by nucleases and facilitate its transmembrane transport and targeted delivery [13]. Common nanocarriers include polymer nanoparticles (such as polyamidoamine dendrimers, PAMAM), liposomes, cationic lipids, and ionizable lipids [[14], [15], [16], [17]]. Among these, PAMAM dendrimers, due to their excellent biocompatibility, modifiability, and efficient gene delivery capabilities, have become an important research focus in the field of RNAi delivery [[18], [19], [20]]. However, the presence of the BBB prevents nearly 100 % of large molecules (such as proteins and nucleic acids) and over 98 % of small molecule therapeutic agents from entering the brain [21]. This limitation severely hinders progress in the treatment of intracranial diseases, including brain tumors, neurodegenerative diseases, and central nervous system infections.

Although specific modifications on the surface of particles can be used to achieve brain targeting and enhance cellular uptake [22], thereby enabling the delivery of drugs across the BBB, the complexity of proteins in the blood leads to the formation of a protein corona around the particles, which can cause targeting failure [23,24]. Additionally, cationic nanoparticles have a short metabolic half-life and strong liver accumulation in the body, which significantly impedes the development and application of effective nanocarrier-based drug delivery systems in clinical settings [[25], [26], [27]].

Focused ultrasound (FUS) assisted microbubbles (MBs) or nanodroplets (NDs) cavitation technology is currently considered one of the safest and most effective methods for temporarily opening the BBB [28]. This technique leverages the interaction between ultrasound waves and MBs or NDs to generate localized mechanical vibrations and cavitation effects, thereby temporarily disrupting the BBB and facilitating drug delivery into the brain [29]. However, this approach still struggles to address the challenges associated with the *in vivo* metabolism of nanoparticles [30]. Although research on drug-loaded MBs and NDs has been published, the hydrophobic nature of the perfluorocarbon core further complicates uniform drug dispersion within the core, resulting in low drug loading efficiency [31]. Additionally, drug loading on the shell may destabilize the MBs and NDs, causing them to rapidly rupture or aggregate *in vivo*, further reducing drug delivery efficiency [[32], [33], [34]]. This has yet to resolve the challenge of siRNA delivery to the brain [35]. Furthermore, studies have shown that traditional FUS transducers are limited by fixed focal size and lack real-time imaging capabilities, relying instead on post-hoc Magnetic Resonance Imaging (MRI) for edema detection and imprecise passive cavitation monitoring (PCM) techniques [36]. To address these limitations, recent advancements in imaging-guided ultrasound systems have emerged as a promising alternative for BBB modulation [37].

**Scheme 1:**
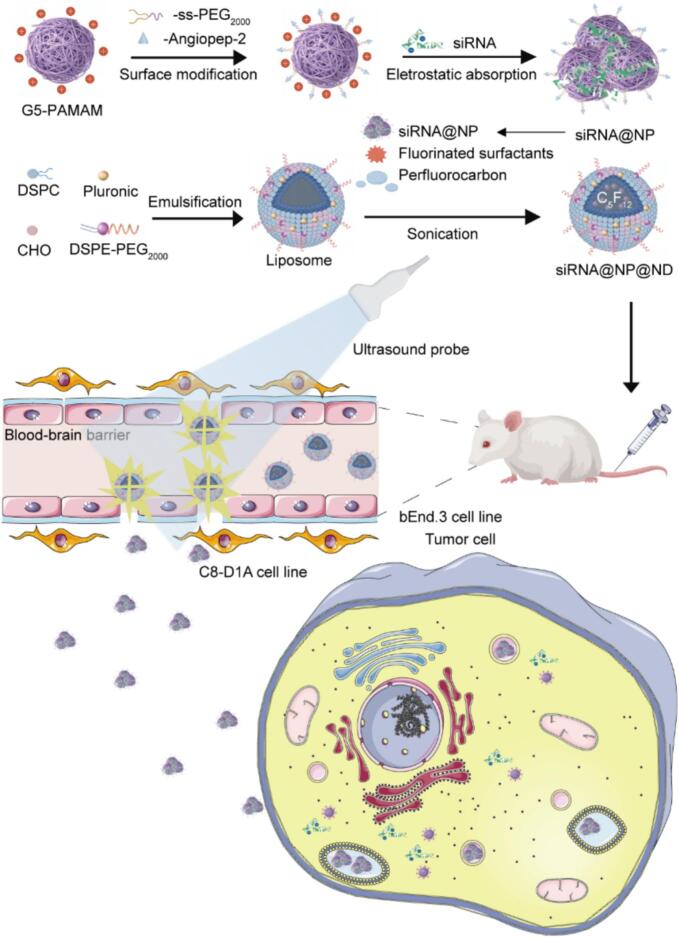
Development and fabrication of siRNA@NP@ND for targeted delivery to brain tumor cells.

In this study, we developed an ultrasound-responsive “cluster bomb” nanoplatform capable of transient BBB opening to facilitate enhanced siRNA delivery into the brain. As illustrated in Scheme 1, this delivery system comprises nanodroplets encapsulating siRNA-loaded nanoparticles. The platform construction involved three key design elements. First, PAMAM dendrimers were selected as optimal siRNA delivery vehicles due to their cationic charge characteristics. Second, surface engineering was implemented through: PEGylation via disulfide linkages to confer glutathione (GSH)-responsive deshielding properties, and functionalization with angiopep-2 ligands to enhance BBB penetration capability, yielding the final siRNA with PAMAM dendrimers (siRNA@NP) construct. Third, through strategic incorporation of fluorinated surfactants, we achieved successful encapsulation of cationic siRNA@NP into charge-neutral perfluorocarbon-based nanodroplets. Subsequently, we thoroughly characterized the cluster bomb and its functionalities *in vitro*. Finally, in conjunction with diagnostic ultrasound [38], we validated its ability to controllably open the BBB and precisely deliver siRNA into the brain both *in vitro* and *in vivo*.

## 2 Results and Discussion

### 2.1 Preparation and characterization of siRNA@NP@ND

In this study, we designed a “cluster bomb” to achieve precise delivery of siRNA to the brain. The preparation process of the cluster bomb consists of two main steps. First, the nanocomplexes of siRNA@NP were prepared using a nanoprecipitation method. The PAMAM dendrimer was modified with disulfide-linked PEG chain for GSH responsiveness and angiopep-2 for BBB penetration. Second, after dispersing the nanoparticles in perfluoropentane using fluorinated surfactants, the ultrasonic emulsification method was employed to fabricate siRNA@NP-loaded nanodroplets. The neutral nanodroplets decrease the non-specific uptake of cationic nanoparticles by the reticuloendothelial system, thereby prolonging the circulation time of siRNA in the bloodstream. This, in turn, enables efficient accumulation at the target site through ultrasound stimulation.

**Fig. 1:**
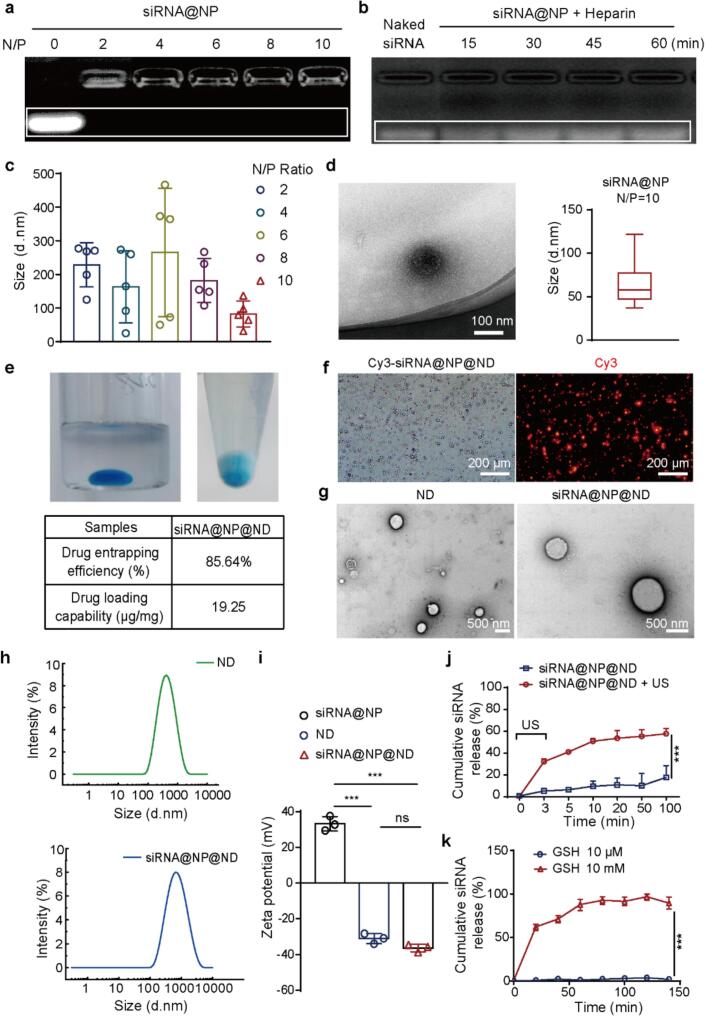
**Characterization of siRNA@NP@ND.** (a) Agarose gel electrophoresis analysis of siRNA@NP at different N/P ratios (0, 2, 4, 6, 8, 10). N/P represents the molar ratio of amine groups in the polymer to phosphate groups in RNA. (b) Gel electrophoresis results of siRNA@NP after incubation with heparin at different time points (15 min, 30 min, 45 min, 60 min). siRNA bands in the gel are highlighted by white boxes. (c) Size distribution of siRNA@NP at varying N/P ratios, with each group analyzed in quintuplicate (n = 5). (d) Transmission electron microscopy (TEM) image of siRNA@NP (scale bar: 100 nm). Particle size distribution of siRNA@NP (88 nanoparticles in total) is shown. (e) Appearance, drug encapsulation efficiency (EE, %), and drug loading capacity (DLC) of siRNA@NP@ND. The upper-left image shows drug-loaded nanoparticles evenly dispersed in perfluorocarbon droplets within the liposomal solution, while the upper-right image depicts precipitated drug-loaded nanodroplets at the bottom with a clear supernatant. (f) Representative bright-field (left) and fluorescence (right) microscopy images of Cy3-siRNA@NP@ND (red: Cy3 fluorescence signal; scale bar: 200 µm). (g) TEM images of ND and siRNA@NP@ND (scale bar: 500 nm). (h) Size distribution of ND and siRNA@NP@ND measured by dynamic light scattering (DLS). (i) Zeta potential values of siRNA@NP, ND, and siRNA@NP@ND determined by DLS (n = 3). (j) Ultrasound-mediated Cy5-siRNA release profile from siRNA@NP@ND (n = 3). (k) The redox-sensitive siRNA release from siRNA@NP *in vitro* (n = 3). Data are presented as mean ± SD. Statistical analysis was performed using one-way ANOVA with Tukey’s post hoc test for multiple comparisons, and paired comparisons were analyzed by Student’s *t*-test. ***p < 0.001; ns: not significant. (For interpretation of the references to colour in this figure legend, the reader is referred to the web version of this article.)

PAMAM achieved siRNA loading through electrostatic adsorption and the binding capacity of PAMAM to siRNA was evaluated using agarose gel electrophoresis. As shown in Fig. 1a, the naked siRNA bands disappeared when the N/P ratio was greater than 2, indicating the nanoparticles could fully load siRNA. Furthermore, Fig. 1c demonstrated that as the N/P ratio increased, the particle size of siRNA@NP showed a gradual decreasing trend. To ensure effective loading of NP by ND and guarantee that NP could penetrate the BBB, we selected siRNA@NP with an N/P ratio of 10 for subsequent studies. The N/P ratio of 10 was selected based on comprehensive considerations. First, this N/P ratio is higher than the critical threshold required for complete siRNA loading, ensuring efficient encapsulation while avoiding leakage and degradation. Second, siRNA@NP formed at N/P = 10 exhibits the smallest average particle size of approximately 66 nm and the most stable size distribution. The smaller size enhances colloidal stability, reduces nonspecific clearance *in vivo*, and promotes blood–brain barrier penetration for brain delivery. Finally, under this condition, the nanoparticles demonstrate a low premature siRNA release rate in simulated physiological environments, indicating excellent drug retention capacity and structural stability. Overall, this choice optimizes particle size, uniformity, and short-term stability, meeting the specific requirements for constructing ultrasound-responsive nanodroplets and brain delivery. The hydrodynamic diameter of siRNA@NP was 82.23 ± 39.09 nm with a real diameter of 66 nm at N/P ratio of 10 in Fig. 1d. Previous studies have indicated that nanoparticles with a size less than 100 nm exhibit better cell transfection efficiency [39]. Meanwhile, Fig. 1b showed that siRNA@NP exhibited significant siRNA dissociation after 15 min of incubation with anionic materials such as heparin, indicating that NP promoted release siRNA in the cytoplasm to exert its targeted effects.

To enhance the stability of nanoparticles *in vivo*, we employed negative nanodroplets for NP encapsulation, addressing the inherent challenge where positively charged PAMAM particles readily bind with serum proteins and subsequently undergo immune system clearance. A Cy5 fluorescent label (visible as blue coloration) was conjugated to the siRNA strands to verify nanoparticle loading within the nanodroplets. As shown in Fig. 1e, siRNA@NP could be dispersed in perfluoropentane by adding fluorophilic surfactants. Post-centrifugation analysis of the siRNA@NP@ND solution revealed characteristic blue coloration in the lower sediment layer. Through process optimization, the drug encapsulation efficiency of ND for siRNA@NP was as high as 85.64 %, with a drug loading capacity of 19.25 μg/mg. Meanwhile, the nanodroplets exhibited a spherical shape and good dispersibility under the microscope in Fig. 1g. Fluorescence microscopy images revealed significant fluorescence on the nanodroplets, indicating successful loading of nanoparticles into the nanodroplets in Fig. 1f. Furthermore, Fig. 1h revealed that the hydrodynamic diameters of unloaded ND and siRNA@NP@ND were 279.80 ± 4.67 nm and 335.93 ± 17.90 nm, respectively. The increased particle size of drug-loaded nanodroplets, attributable to NP incorporation, but did not affect the microstructure and distribution trend of NDs. Importantly, while siRNA@NP nanoparticles exhibited a positive surface charge with a zeta potential of 33.27 ± 4.04 mV, unloaded ND and siRNA@NP@ND showed a negative surface charge with zeta potentials of −31.04 ± 2.84 mV and −36.32 ± 2.15 mV in Fig. 1i, respectively. The NDs successfully masked the positive charge on the surface of siRNA@NP, providing a crucial assurance for improving circulation time *in vivo*.

Additionally, the nanodroplets could rapidly respond to ultrasound stimulation, and release the siRNA@NP in Fig. 1j. Moreover, 10 mM GSH was used to mimic an intracellular reductive environment [40] to verify the functionality of siRNA@NP in releasing siRNA in response to the microenvironment. Fig. 1k showed that siRNA@NP exhibited significant siRNA release at a 10 mM GSH concentration, with 95 % of siRNA released from siRNA@NP after 100 min of incubation. In contrast, no detectable release was observed in the normal tissue environment (10 µM GSH). Additionally, Fig. S1 showed that the siRNA@NP@ND maintained good size stability at 4 °C, meeting storage requirements. The particle size of the siRNA@NP@ND increased over time but still met usage requirements at 37 °C. Furthermore, the nanodroplets in flowing solution and fixed agar were more directly observed the response under ultrasound stimulation. As shown in Fig. S2, before and after ultrasound stimulation, we could clearly observe the vaporization and concentration reduction of the nanodroplets. Fig. S3 showed that as the imaging ultrasound pressure was adjusted, the bright signal in the ultrasound images gradually increased, indicating significant vaporization and bursting of siRNA@NP@ND. These results demonstrated the feasibility of siRNA@NP@ND releasing its payload under ultrasound stimulation.

### 2.2 Cell uptake and toxicity of siRNA@NP@ND *in vitro*

**Fig. 2:**
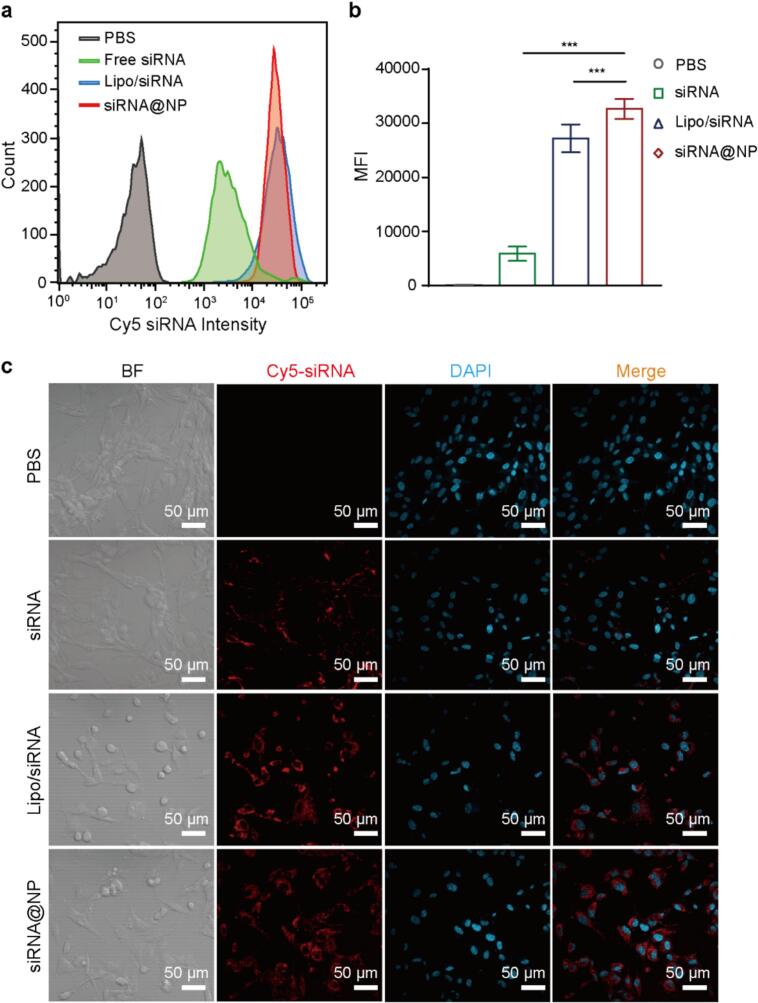
**Cellular internalization efficiency of siRNA@NP sequence.** (a) Flow cytometry analysis of Cy5-siRNA uptake efficiency in U87-MG cells across different treatment groups (PBS, free siRNA, Lipo/siRNA, and siRNA@NP). (b) Mean fluorescence intensity (MFI) of Cy5-siRNA in each treatment group (n = 5). (c) Confocal laser scanning microscopy (CLSM) images showing Cy5-siRNA uptake in U87-MG cells for each treatment group (PBS, free siRNA, Lipo/siRNA, and siRNA@NP). Red: Cy5-siRNA fluorescence; blue: cell nuclei (DAPI staining); scale bar: 50 µm. Bright-field (BF) images are included. Data are presented as mean ± SD. Statistical analysis was performed using one-way ANOVA with Tukey’s post hoc test for multiple comparisons. ***P < 0.001. (For interpretation of the references to colour in this figure legend, the reader is referred to the web version of this article.)

Effective cellular uptake is a prerequisite for siRNA@NP to exert therapeutic effects within cells. Therefore, siRNA labeled with Cy5 was used to evaluate the cellular uptake efficiency of siRNA@NP *in vitro* using flow cytometry and confocal laser scanning microscopy (CLSM). As shown in Fig. 2a, b, compared to naked Cy5-siRNA, the Cy5-siRNA@NP showed stronger fluorescence signals, indicating a more pronounced ability to induce cellular uptake. Additionally, the Cy5-siRNA@NP exhibited significantly stronger fluorescence than Lipo/siRNA, a commonly used as positive control in research. This strongly confirms that Cy5-siRNA@NP possesses excellent cellular uptake capabilities. Furthermore, CLSM results further supported this trend, showing higher fluorescence in the cytoplasm of U87-MG cells for Cy5-siRNA@NP compared to the groups of naked Cy5-siRNA and Lipo/siRNA in Fig. 2c. These results indicate that the engineered siRNA@NP nanocarrier system effectively facilitates intracellular delivery of therapeutic siRNA payloads in U87-MG cells.

Cytotoxicity assessment using CCK-8 assays revealed that siRNA@NP@ND exhibited excellent biocompatibility, showing no detectable cytotoxicity (cell viability > 95 %) even at high siRNA concentrations (1000 nM) in both endothelial and tumor cells (Fig. S4c, d). In contrast, siRNA@NP demonstrated potent dose-dependent therapeutic effects, significantly reducing U87-MG cell viability at 100 nM after 24 h incubation (Fig. S4a, b). This therapeutic efficacy is attributed to the cationic siRNA@NP's superior cellular transfection efficiency and cationic toxicity inherently associated with the dendrimers [41]. Notably, nanodroplet encapsulation of siRNA@NP effectively mitigated its inherent cytotoxicity while maintaining therapeutic potential, demonstrating the system's dual capability for toxicity reduction and therapeutic payload delivery.

### 2.3 *In vitro* safety analysis of Ultrasound-Induced BBB opening

**Fig. 3:**
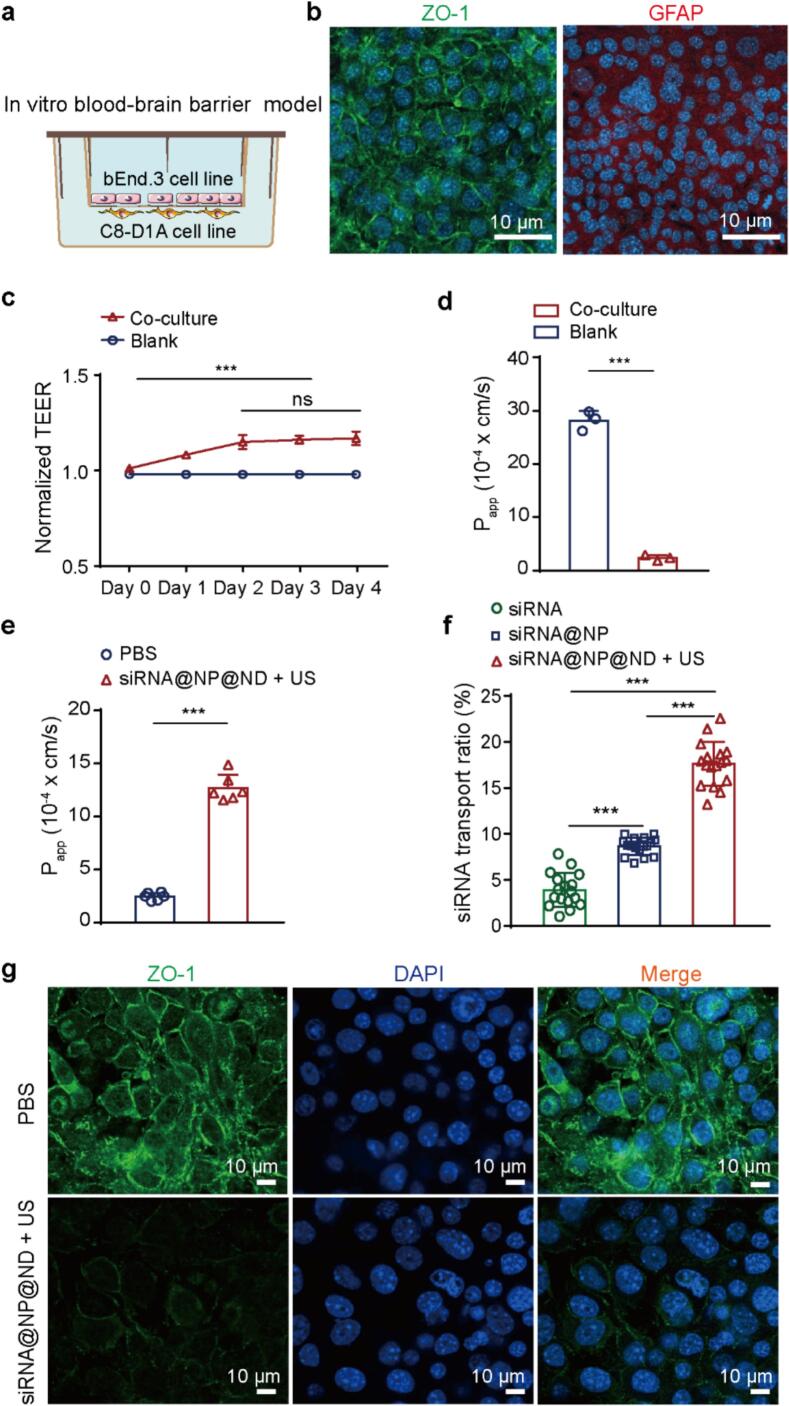
***In vitro* blood**–**brain barrier transport efficiency.** (a) Schematic illustration of a novel high-fidelity *in vitro* BBB model. The upper layer is seeded with mouse brain endothelial cells (bEnd.3), and the lower layer is seeded with mouse brain astrocytes (C8-D1A). (b) Confocal microscopy images of the bilayer cell structure. Left: Endothelial cell layer showing green fluorescence for tight junction protein ZO-1. Right: Astrocyte layer showing red fluorescence for glial fibrillary acidic protein (GFAP) and blue fluorescence for nuclei (DAPI staining). Scale bar: 10 μm. (c) Dynamic changes in transendothelial electrical resistance (TEER) of the co-culture model over 4 days (n = 3). (d) Apparent permeability coefficient (P_app_) of FITC-dextran in the co-culture model on day 4 (n = 3). (e) P_app_ values post-treatment with ultrasound combined with siRNA@NP@ND (n = 6). (f) siRNA transport ratio across the BBB model after ultrasound and siRNA@NP@ND treatment (n = 17). (g) Confocal microscopy visualization of tight junction protein ZO-1 expression in the *in vitro* BBB model for different treatment groups (control and ultrasound + siRNA@NP@ND). Scale bar: 10 μm. Data are presented as mean ± SD. Statistical analysis was performed using one-way ANOVA with Tukey’s post hoc test for multiple comparisons, and paired comparisons were analyzed by Student’s *t*-test. ***p < 0.001; ns: not significant. (For interpretation of the references to colour in this figure legend, the reader is referred to the web version of this article.)

Next, we inoculated bEnd3 and C8-D1A cells in the upper and lower chambers of transwell inserts, respectively, to construct a double-layer BBB model to evaluate ultrasound-enhanced BBB permeability in Fig. 3a. As shown in Fig. 3c, transepithelial electrical resistance (TEER) values during barrier maturation remained stable between days 2–4, ‌significantly higher than those in the non-barrier group‌. Quantitative validation through apparent permeability (P_app_) measurements in Fig. 3d revealed 91.6 % reduction in FITC-dextran flux across the bilayer system compared to acellular controls at day 4 (2.37 ± 0.45 × 10^-4^ cm/s vs. 28.17 ± 1.49 × 10^-4^ cm/s, Fig. 3d), confirming functional BBB integrity. Additionally, as depicted in Fig. 3b, confocal microscopy images of the bilayered cell structure demonstrate clear expression of the tight junction protein ZO-1 between endothelial cells. Beneath the endothelial layer, densely arranged C8-D1A cells (characterized by GFAP expression) are observed, further indicating the integrity of the bilayer BBB model.

Furthermore, we first investigated the BBB-modulating efficacy of ultrasound-activated empty nanodroplets using our established bilayer model. As demonstrated in Fig. S5, progressive increase in ultrasound stimulation time (1–5 min) induced dose-dependent architectural reorganization of the BBB cellular interface. High-energy sonication parameters (20 % duty cycle, 3 W acoustic power, 1 MHz frequency, 5 min duration) triggered significant structural disintegration (white arrow) of the BBB.

To systematically investigate ultrasound-mediated BBB modulation while minimizing vascular morphological alterations, we selected intermediate sonication parameters (20 % duty cycle, 3 W acoustic power, 1 MHz frequency, 3 min duration) for mechanistic exploration. As shown in Fig. 3e, ultrasound-triggered nanodroplet vaporization significantly enhanced paracellular permeability in bilayer BBB models (P_app_ increased 4.15-fold), confirming the universal efficacy of this strategy across experimental systems. Mechanistically, immunoblot analysis (Fig. 3g) revealed a marked reduction in tight junction protein ZO-1 expression post-sonication (vs. PBS), suggesting ultrasound-activated nanodroplets induce reversible barrier opening primarily through tight junction reorganization rather than permanent endothelial damage [42].

Subsequently, to systematically evaluate the BBB-penetrating efficacy of the siRNA@NP@ND system under ultrasound activation, we conducted quantitative comparisons across three delivery modalities (Fig. 3f). Naked siRNA exhibited baseline permeability, while siRNA@NP demonstrated a 1.21-fold enhancement in *trans*-BBB transport efficiency. Remarkably, siRNA@NP@ND further improved permeability by 1.03-fold over siRNA@NP, achieving a cumulative 3.50-fold increase compared to free siRNA, confirming the critical role of acoustic activation in payload release and barrier modulation.

### 2.4 Reversible opening of BBB using siRNA@NP@ND

**Fig. 4:**
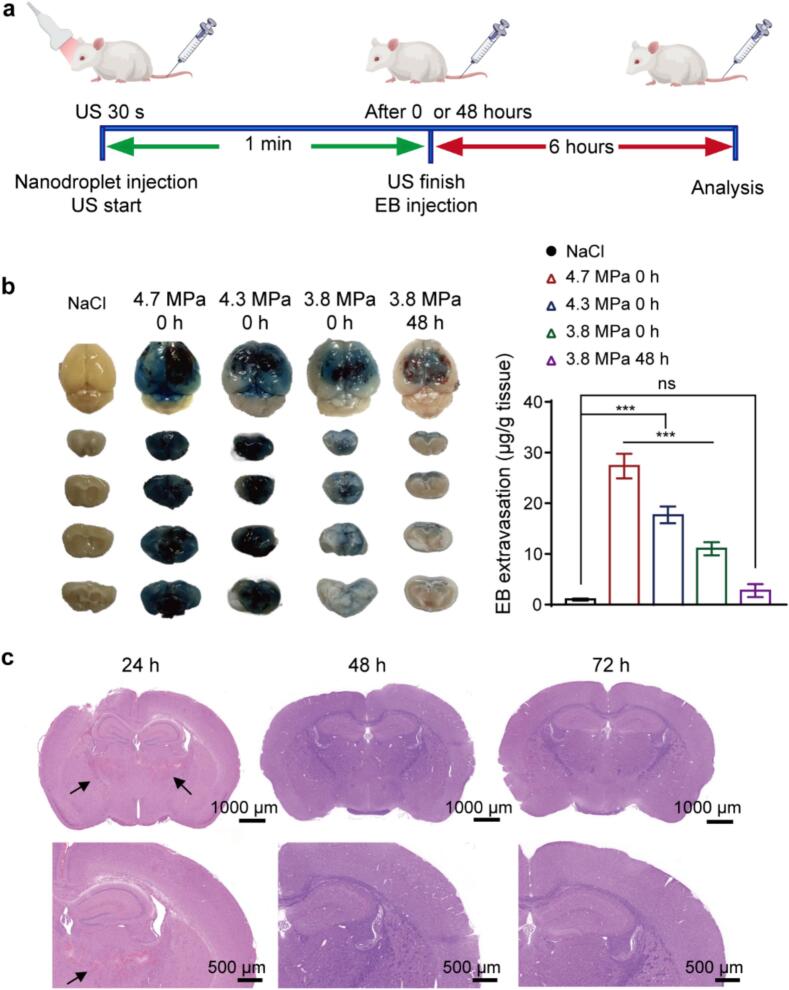
***In vivo* investigation of blood–brain barrier disruption.** (a) Schematic illustration of ultrasound (US)-mediated BBB opening combined with siRNA@NP@ND. Evans Blue (EB) permeation in the brain was used to assess BBB disruption. (b) Spatial distribution and quantitative analysis of Evans Blue (EB) extravasation in coronal brain sections of mice (n = 3). (c) Hematoxylin and eosin (H&E) staining of mouse brain sections at different time points post-ultrasound intervention (24 h, 48 h, 72 h). Hemorrhage (indicated by black arrows) was observed at 24 h but resolved by 48 h. Whole-brain (scale bar: 1000 μm) and localized brain regions (scale bar: 500 μm) are shown for each group. Data are presented as mean ± SD. Statistical analysis was performed using one-way ANOVA with Tukey’s post hoc test for multiple comparisons. ***p < 0.001; ns: not significant. (For interpretation of the references to colour in this figure legend, the reader is referred to the web version of this article.)

To optimize ultrasound-mediated BBB disruption parameters *in vivo*, we employed Evans blue (EB) extravasation as a quantitative biomarker for barrier permeability. Following ‌tail vein administration‌ of ‌siRNA@NP@ND‌ (2 mg/kg siRNA equivalent), focused ultrasound (4 MHz central frequency, varying acoustic pressures) was applied to the ‌tumor-prone regions in the right hemisphere‌ for 1 min guided by ultrasound imaging (Fig. 4a). EB (4 % w/v, 4 mL/kg) was intravenously administered at staggered timepoints post-sonication (0, 48 h), with terminal perfusion and brain harvesting conducted 6 h post-EB injection. As demonstrated in Fig. 4b, control groups without ultrasound exposure exhibited minimal Evans blue (EB) extravasation (1.02 ± 0.16 μg EB/g brain tissue), confirming intact BBB integrity. Progressive elevation of acoustic pressure (3.8–4.7 MPa) induced dose-dependent EB accumulation in murine brains, with significant enhancement observed at therapeutic parameters (3.8 MPa: 11.04 ± 1.14 μg/g; 4.3 MPa: 17.71 ± 1.37 μg/g; 4.7 MPa: 27.34 ± 1.99 μg/g). This phenomenon is attributed to cavitation effects generated during ultrasound-mediated nanodroplet (ND) vaporization, which transiently disrupts tight junctions and increases paracellular permeability to small molecules like EB [43].

Further evaluation at 3.8 MPa acoustic pressure revealed time-dependent BBB recovery dynamics. Evans blue (EB) extravasation decreased by 74.83 % at 48 h post-sonication compared to immediate (0 h) EB administration, confirming the transient/reversible nature of ultrasound-mediated BBB opening. To balance therapeutic efficacy and biosafety, we conducted longitudinal safety profiling at 3.8 MPa (mechanical index = 1.3). As shown in Fig. 4c, after 24 h of ultrasound intervention, the H&E staining results clearly indicated local hemorrhaging. However, as time progressed, at 48 h and 72 h, there was a noticeable recovery from the local hemorrhaging. This observation indicates that while some initial damage may occur, the brain demonstrates a significant capacity to recover over time, thereby supporting the safety profile of the intervention system.

### 2.5 Pharmacokinetics and biodistribution of siRNA@NP@ND

To evaluate the brain-targeting efficacy of Angiopep-2 peptide modification on siRNA@NP, we prepared Cy5-labeled siRNA constructs (Cy5-siRNA@PSP and Cy5-siRNA@PSPA) for real-time fluorescence imaging in BALB/c mice. *In vivo* fluorescence imaging at 2 h, 4 h, and 6 h post-injection revealed that Cy5-siRNA@PSPA (Angiopep-2 modified) exhibited progressive fluorescence accumulation in brain tissue in Fig. S6. After 6 h dissection, the fluorescence signal intensity in the siRNA@PSPA group was 1.98 times higher than that in the non-targeted siRNA@PSP group. Consistent with established literature, Angiopep-2 may facilitate this enhancement by specifically targeting the low-density lipoprotein receptor-related protein 1 (LRP-1), which is highly expressed on brain endothelial cells and mediates nanocarrier transcytosis [[44], [45], [46]]. Our findings align with prior reports, suggesting Angiopep-2 functionalization effectively enhances delivery efficiency to the brain.

Simultaneously, the ability of nanodroplets to prevent premature immune capture of siRNA@NP is critical for successful brain drug delivery, prompting us to investigate the pharmacokinetics of siRNA, siRNA@NP, and siRNA@NP@ND following single intravenous administration in tumor-free BALB/c mice. As shown in Fig. S7, siRNA@NP@ND exhibited significantly prolonged systemic circulation with a blood half-life of 37.1 min, outperforming siRNA@NP (14.5 min) and free siRNA (12.7 min). This enhanced circulation stability is attributed to the electronegative surface of nanodroplets shielding the cationic siRNA@NP from immune recognition, while the siRNA@NP core protects siRNA from nuclease degradation. Additionally, the optimized 80 nm particle size prevents rapid renal clearance. These findings collectively demonstrate the effectiveness of the nanodroplet platform in enhancing systemic circulation time, establishing its potential for improved brain-targeted delivery.

### 2.6 Ultrasound-mediated nanodroplets for efficient siRNA delivery to the brain

**Fig. 5:**
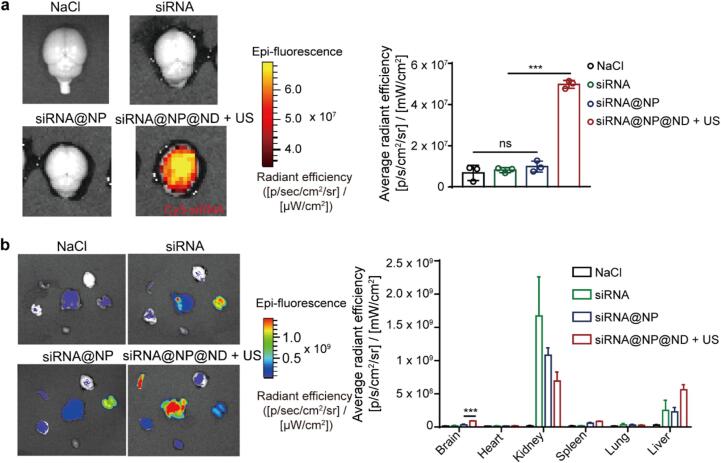
***In vivo* analysis of brain-targeted siRNA delivery and biodistribution.** (a) *Ex vivo* imaging and fluorescence quantification of Cy5-siRNA (0.5 mg/kg dose) in brain tissues from mice treated with different groups: NaCl (control), free siRNA, siRNA@NP, and siRNA@NP@ND + US (n = 3). Red signals indicate Cy5-siRNA accumulation. (b) *Ex vivo* imaging and fluorescence quantification of Cy5-siRNA (0.5 mg/kg dose) in major organs (brain, heart, liver, spleen, lungs, and kidneys) from mice across treatment groups: NaCl, free siRNA, siRNA@NP, and siRNA@NP@ND + US (n = 3).Data are presented as mean ± SD. Statistical analysis was performed using one-way ANOVA with Tukey’s post hoc test for multiple comparisons. ***p < 0.001; ns: not significant. (For interpretation of the references to colour in this figure legend, the reader is referred to the web version of this article.)

To validate the ultrasound-mediated brain delivery capability of siRNA@NP@ND, we intravenously administered different drug carriers and performed imaging-guided ultrasound stimulation (1 MHz, 3.8 MPa, 1 min) on the siRNA@NP@ND group. Two hours post-stimulation, mice underwent cardiac perfusion followed by *ex vivo* brain fluorescence imaging analysis. As shown in Fig. 5a, the siRNA@NP@ND + US group exhibited a significant increase in Cy5 fluorescence signal intensity compared to the siRNA@NP group. This improvement is attributed to the synergistic effects of ultrasound-triggered nanodroplet vaporization and transient BBB opening, which facilitated efficient siRNA extravasation into brain parenchyma.

The biodistribution analysis of siRNA in Fig. 5b revealed that the siRNA@NP@ND + US group significantly altered the systemic distribution profile, reducing renal accumulation compared to free siRNA while increasing hepatic and splenic uptake. Despite the enhanced brain accumulation, the majority of the administered dose was still localized in the liver. This distribution pattern highlights the dual role of the liver and spleen as primary clearance organs for nanodroplet systems, while confirming the ability of ultrasound-mediated delivery to redirect a therapeutically significant fraction of siRNA to the brain.

**Fig. 6:**
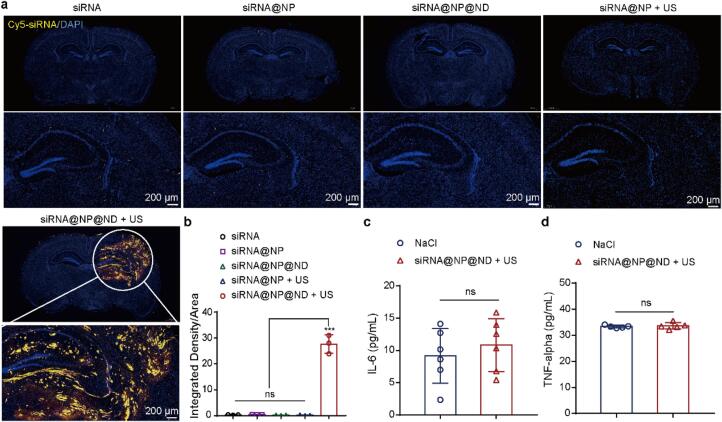
***In vivo* evaluation of brain-targeted siRNA delivery efficacy and safety.** Distribution (a) and quantitative analysis (b) of Cy5-siRNA (0.5 mg/kg dose) in the brains of normal mice across treatment groups: free siRNA, siRNA@NP, siRNA@NP@ND, siRNA@NP + ultrasound (US), and siRNA@NP@ND + US (n = 3). Yellow signals indicate Cy5-siRNA, blue signals represent nuclei (DAPI staining), and white boxes outline the ultrasound-targeted regions. Scale bar for images below each group: 200 µm. (c.d) Cytokine levels in mice treated with NaCl (control) or US + siRNA@NP@ND (n = 5–6).Data are presented as mean ± SD. Statistical analysis was performed using one-way ANOVA with Tukey’s post hoc test for multiple comparisons, and paired comparisons were analyzed by Student’s *t*-test. ***p < 0.001; ns: not significant. (For interpretation of the references to colour in this figure legend, the reader is referred to the web version of this article.)

To further investigate siRNA accumulation in the brain, we performed *ex vivo* brain coronal sectioning and fluorescence imaging. As shown in Fig. 6a. the results demonstrated that siRNA@NP@ND combined with ultrasound stimulation significantly enhanced brain delivery efficiency compared to other groups. Intravenous injection of free siRNA, due to its short circulation half-life (12.7 min) and rapid enzymatic degradation, failed to achieve detectable brain accumulation. While siRNA@NP, protected from enzymatic degradation and enhanced by Angiopep-2-mediated targeting (Fig. S6), showed increased accumulation in cerebral vasculature, post-perfusion analysis revealed minimal parenchymal penetration, highlighting its limited BBB penetration capability. Similarly, siRNA@NP + US exhibited negligible brain tissue accumulation, as siRNA@NP alone does not respond to ultrasound stimulation. Additionally, siRNA@NP@ND without ultrasound stimulation also demonstrated minimal brain delivery. In contrast, the siRNA@NP@ND + US group exhibited localized fluorescence signals exclusively in ultrasound-irradiated regions, achieving a 70.8-fold increase in fluorescence intensity compared to siRNA@NP alone in Fig. 6b. This targeted enhancement is attributed to ultrasound-triggered nanodroplet vaporization, which simultaneously releases siRNA@NP and transiently opens the BBB, enabling localized and efficient brain delivery. The spatial specificity of this approach, with fluorescence signals confined to ultrasound-irradiated regions, underscores its precision in achieving regional brain delivery while minimizing off-target effects.

Compared to alternative brain delivery strategies such as intranasal administration or convection-enhanced delivery, which often lack spatial selectivity and carry risks of systemic neurological adverse effects, ultrasound-mediated nanodroplet BBB opening offers a targeted and controllable approach. This method relies on mechanical disruption of tight junction proteins, with the extent of BBB opening and associated brain injury directly correlated with ultrasound energy levels. As demonstrated in Fig. 4, transient brain injury and microhemorrhages induced by ultrasound-triggered nanodroplet vaporization are fully reversible within 72 h, with complete restoration of BBB integrity, these bioeffects are confined to ultrasound-irradiated regions, enabling precise control over both the degree of brain injury and drug delivery efficiency. This spatial specificity minimizes off-target exposure and associated neurological risks, while allowing for tailored therapeutic outcomes based on acoustic parameter optimization. The ability to balance therapeutic efficacy with controlled, reversible bioeffects positions ultrasound-mediated nanodroplet delivery as a superior strategy for targeted brain therapeutics, addressing the limitations of conventional approaches that often compromise safety for delivery efficiency.

Additionally, Fig. S8 results showed that compared to the control group, ultrasound-activated droplet treatment did not cause significant damage to organs such as the heart, liver, spleen, lungs, and kidneys, further demonstrating the good biological safety of ultrasound combined with nanodroplet treatment.

To comprehensively evaluate the *in vivo* safety profile of the siRNA@NP@ND + US therapeutic system, we collected blood samples from different treatment groups and performed hematological, biochemical, and immunological analyses. As shown in the Fig. 6c and 6d, key inflammatory markers including IL-6 (acute phase response and immune regulation) and TNF-α (inflammation initiation and apoptosis) showed no significant changes in the siRNA@NP@ND + US group compared to saline controls (IL-6: 10.82 ± 3.74 pg/mL vs. 9.16 ± 3.87 pg/mL; TNF-α: 33.64 ± 1.09 pg/mL vs. 33.34 ± 0.46 pg/mL). Furthermore, Fig. S9 demonstrates that siRNA@NP@ND treatment did not induce abnormal hematological parameter changes, with all measured indices—including WBC, MID, GRA, UREA, LYM, RBC, PLT, HCT, GLU, MCH, etc., remaining within normal physiological ranges. These findings collectively indicate that the siRNA@NP@ND + US system exhibits excellent biosafety without inducing significant acute toxicity in mice, supporting its potential for further clinical translation.

In summary, this study demonstrates that siRNA@NP@ND combined with imaging-guided ultrasound enables safe and localized BBB opening and efficient brain drug delivery. The delivery efficiency and *in vivo* safety profile are critically dependent on key parameters including ultrasound intensity, sonication duration, nanodroplet concentration, and drug loading capacity. While our nanodroplet-encapsulated nanoparticle system achieves localized and efficient siRNA delivery to the brain, it is accompanied by transient microhemorrhages that, although reversible within 72 h (Fig. 4), present a safety concern. Future studies should focus on optimizing nanodroplet size (50–150 nm) and ultrasound parameters (burst length, duty cycle, mechanical index) to identify the therapeutic window that balances delivery efficiency (target: >5% brain accumulation) with minimized hemorrhagic risk. Additionally, advanced ultrasound modalities such as pulsed-wave sonication or frequency modulation could further enhance spatial precision and safety, paving the way for clinical translation of this targeted brain delivery platform.

## 3 Conclusion

In summary, our study developed a novel ultrasound-responsive cluster bomb (siRNA@NP@NDs) that not only protects siRNA@NP from being captured by immune organs, thereby prolonging its circulation time, but also releases siRNA@NP at the target site under ultrasound stimulation. More importantly, the energy generated by NDs bursting can simultaneously open the BBB, and the Angiopep-2 modification on the NP surface further enhances its brain targeting and penetration capabilities, thereby increasing the delivery efficiency of siRNA to the brain. This dual-layered design elegantly resolves the conflicting requirements of systemic circulation (stealth properties) and brain delivery (active targeting), achieving a 70.8-fold increase in brain accumulation compared to siRNA@NP. Although the ultrasound-guided siRNA@NP@ND delivery system shows promise in preliminary animal studies, its clinical translation faces several challenges including insufficient systematic assessment of long-term safety and immunogenicity, lack of standardized and personalized ultrasound parameter protocols, and unverified cross-species applicability/stability of targeting ligands (e.g., Angiopep-2) in human models. Future optimizations will focus on improving nanodroplet shell formulation to prolong circulatory stability and reduce nonspecific organ uptake, thereby enhancing brain-specific drug accumulation. Moreover, scalable manufacturing and Good Manufacturing Practice (GMP) compatibility of the nanoparticle-nanodroplet complex require optimization along with enhanced operational feasibility in clinical settings. More importantly, validation in more human-relevant BBB models and exploration of combination therapies with standard treatments such as temozolomide or immunotherapies are needed to fully evaluate clinical potential. Additionally, future work must address batch-to-batch reproducibility in the fabrication of siRNA@NP@NDs, potentially via microfluidic technology, and establish lyophilization protocols suitable for clinical storage. Furthermore, to our knowledge, this represents the first reported use of nanodroplet-encapsulated nanoparticles for siRNA delivery, with demonstrated potential for treating glioblastoma. The modular design of this platform allows for adaptation to other therapeutic payloads (e.g., mRNA, CRISPR-Cas9) and disease targets (e.g., neurodegenerative disorders, brain metastases), offering a versatile strategy for precision medicine applications.

## 4 Experimental section

All materials, methods, and additional data can be found in the Electronic Supplementary Material.

Electronic Supplementary Material

Supplementary Material (including the experimental section, the illustration of synthetic process for siRNA@NP@ND, the biocompatibility of siRNA@NP@ND, acoustic pressure and H&E staining of main organs studies) is available in the online version of this article.

## CRediT authorship contribution statement

**Tianyu Guo:** Writing – review & editing, Writing – original draft, Visualization, Validation, Project administration, Methodology, Investigation, Formal analysis, Data curation, Conceptualization. **Feihong Dong:** Writing – review & editing, Writing – original draft, Visualization, Validation, Project administration, Methodology, Investigation, Formal analysis, Data curation, Conceptualization. **Jingyi Yin:** Visualization, Validation, Software. **Xinnan Wang:** Visualization, Validation, Data curation. **Pengting Min:** Visualization, Validation, Data curation. **Jiabin Zhang:** Visualization, Validation, Software. **Heping Cheng:** Writing – review & editing, Supervision, Resources, Project administration, Funding acquisition. **Jue Zhang:** Visualization, Validation, Software.

## Declaration of competing interest

The authors declare that they have no known competing financial interests or personal relationships that could have appeared to influence the work reported in this paper.
