# Supplementary material for: A novel ultrasound-responsive cluster bomb system for efficient siRNA delivery in brain
Source: Ultrason Sonochem. 2025 Jun 25;120:107446. doi: 10.1016/j.ultsonch.2025.107446 (PMC12268195; doi:10.1016/j.ultsonch.2025.107446)
Supplement: Supplementary Data 1 [file mmc1.docx]

**A Novel Ultrasound-Responsive Cluster Bombs System for Efficient siRNA Delivery in Brain**

*Tianyu Guo^1,6^, Feihong Dong^1,6^*, Jingyi Yin^2^, Xinnan Wang^2^, Pengting Min^2^, Jiabin Zhang^1^, Heping Cheng^1,4,5^, Jue Zhang^2,3,4^**

^1^ State Key Laboratory of Membrane Biology, National Biomedical Imaging Center, Peking-Tsinghua Center for Life Sciences, Institute of Molecular Medicine, College of Future Technology, Peking University, Beijing, 100871, China.

^2^ Academy for Advanced Interdisciplinary Studies, Peking University, Beijing, 100871, China.

^3^ College of Engineering, Peking University, Beijing, 100871, China.

^4^ National Biomedical Imaging Center, Peking University, Beijing, 100871, China.

^5^ Research Unit of Mitochondria in Brain Diseases, Chinese Academy of Medical Sciences, PKU-Nanjing Institute of Translational Medicine, Nanjing, 211899, China

^6^ These authors contributed equally to this work

* Correspondence should be addressed to Feihong Dong ([dongfeihong@pku.edu.cn](mailto:zhangjue@pku.edu.cn)), Jue Zhang (zhangjue@pku.edu.cn).

**Materials and methods**

**Materials**

PAMAM-ss-PEG_2000_ and PAMAM-ss-PEG_2000_-Angiopep-2 were synthesized by Xi'an Ruixi Biological Technology Co., Ltd (Xi'an, China). DSPC and DSPE-PEG_2000_ were purchased from Xi'an Ruixi Biological Technology Co., Ltd (Xi'an, China). Negative control (siNC) was synthesized by GenePharma (Shanghai, China). Pluronic, Perfluoropentane (PFP) and CHO were purchased from Aladdin. L-Glutathione (GSH) was purchased from J&K Scientific Ltd. 1 x Hoechst 33,342 (stain Solution) SL7130 was purchased from Coolaber (Beijing, China). 0.9% saline (w/v, 0.9 g NaCl/100 mL water) were purchased from Biotopped (Beijing, China). Lipofectamine™ RNAiMAX Transfection Reagent, ZO-1 antibody, TNF-α ELISA Kit, and IL-6 ELISA Kit were purchased from ThermoFisher. All other chemicals used in the laboratory, including ethanol and glycerol, were of analytical reagent grade and were used without further purification. BALB/c female mice were purchased from Beijing Charles River Experimental Animal Technology. bEnd.3 cells and C8-D1A cells were purchased were obtained from American Type Culture Collection (ATCC). All animal experimental protocols were reviewed and approved by the local animal care committee of Peking University (AAIS-ZhangJ-9).

**Preparation of siRNA@NP@ND**

Nanoparticles were prepared via a nanoprecipitation method. Briefly, PAMAM-SS-PEG-ANGIOPEP-2 was weighed and dissolved in DEPC water, and Cy5-siRNA synthesized by GenePharma was also weighed and dissolved in DEPC water. After mixing the two solutions and allowing them to stand for 30 minutes, an siRNA@NP solution with different N/P ratio was formed. Further, drug-loaded nanodroplets were prepared using the thin-film emulsification and ultrasonic emulsification methods. Briefly, DSPC, CHO, DSPE-PEG_2000_, and Pluronic (mass ratio 25:11:10:11) were weighed and dissolved in ethanol, then placed in a round-bottom flask. A thin film was formed by high-temperature vacuum evaporation using a rotary evaporator, followed by hydration with aqueous solution (80% DEPC water, 10% glycerol, 10% propylene glycol) in the flask. PFP, siRNA@NP with N/P ratio of 10, and fluorinated oil (Krytox 157 FSL) were thoroughly mixed (volume ratio 20:10:3) to form a homogeneous mixture, which was then added to the the above solution at a volume ratio of 33:1000. The combined solution was processed using an ultrasonic emulsification device to form the siRNA@NP@ND solution. The resulting solution was centrifuged to remove unencapsulated components from the supernatant, and the pellet was resuspended in PBS for storage at 4°C.

**Characterization of Nanodroplet and nanoparticle**

Particle size distribution, zeta potential data and stability were obtained using the Zetasizer Nanosystem (Omni, Brookhaven). The samples were prepared by diluting the initial solution to appropriate concentration with deionized water. These samples were placed in a measurement cell and analyzed at 25°C. All DLS measurements were performed in triplicate. Morphological features were observed using a transmission electron microscope (Tecnai T20, FEI). A drop of the emulsion was applied to a carbon-coated copper grid and allowed to equilibrate for 2 minutes. The grid was then stained with a 3% ammonium molybdate solution. Excess liquid at the grid edges was gently removed by briefly touching filter paper, and the grid was dried at room temperature before being transferred to the TEM operating at an accelerating voltage of 200 kV.

**Encapsulation efficiency**

The encapsulation efficiency of siRNA within siRNA@NP was evaluated via agarose gel electrophoresis. Samples of siRNA@NP at various N/P ratios, along with free siRNA, were loaded onto a 2% agarose gel and visualized using a Gel Imaging System. Meanwhile, siRNA labeled with Cy5 was used to evaluate the loading efficiency of nanodroplets. The drug-loaded nanodroplets (Cy5-siRNA@NP@ND) were subjected to high-speed centrifugation, and the supernatant was collected and transferred to a 96-well plate. Cy5 fluorescence intensity was measured using a microplate reader (excitation: 645 nm, emission: 670 nm), with a corresponding Cy5-siRNA standard curve generated concurrently. The Cy5-siRNA drug entrapment efficiency and drug loading efficiency were calculated using the following equation:

*Drug entrapment efficiency (%) =*$\frac{loaded amout of drug}{Total amount of drug added}\times100$ [1]

*Drug loading capacity =*$\frac{Loaded amount of drug}{amount of lipid+loaded amount of drug}$ [2]

***In vitro* drug release**

siRNA@NP was mixed with 10 mM and 10 μM GSH solutions and incubated at 37°C for predetermined time points (0, 20, 40, 60, 80, 100, 120, and 140 minutes). The mixture was then centrifuged using a centrifugal filter tube (MWCO: 30 kDa) at 13,000 g for 15 minutes, and the filtrate was transferred to 96-well plates for Cy5 fluorescence analysis (excitation: 645 nm, emission: 670 nm) using a microplate reader. In a separate experiment, the prepared Cy5-siRNA@NP@ND were divided into two groups. One group underwent ultrasonic irradiation (1 W/cm², 60 seconds, 1.0 MHz) using a probe (UT1041, Dundex), while the other served as the control. At fixed time intervals, the fluorescence intensity of Cy5 siRNA was measured, and the corresponding concentration was determined using a standard curve. The cumulative release rate (CER) was calculated using the formula: (CER= released Cy5 siRNA / Initially encapsulated Cy5 siRNA in the droplets) × 100%.

**Cell uptake by flow cytometry**

U87-MG cells were seeded in 6 well plates at a density of 1.5×10^5^ cells per well and cultured overnight in DMEM medium. The old medium was then removed and replaced with fresh medium. PBS, free Cy5 siRNA, Lipo/Cy5-siRNA and Cy5-siRNA@NP were added separately to achieve a final drug concentration of 100 nM. The cells were incubated for 2 hours at 37°C in a 5% CO₂ environment. After treatment, the medium was removed, and the cells were washed three times with PBS, followed by trypsin digestion, centrifugation, and resuspension in fresh PBS. All samples were analyzed using a flow cytometer (LSRFortessa, Becton Dickinson).

**Cell uptake by confocal laser scanning microscopy**

To visualize the uptake of free Cy5‑siRNA and Cy5‑labeled siRNA@NP in U87‑MG cells, the cells were seeded in confocal dishes at a density of 1.5×10^5^ cells per dish and cultured overnight. The old medium was removed and replaced with 450 μL of fresh medium. Then, 50 μL of PBS, free Cy5‑siRNA, or Cy5‑labeled siRNA@NP was added to achieve a final siRNA concentration of 100 nM. The cells were incubated at 37°C in a 5% CO₂ atmosphere for 2 hours. Subsequently, the cells were washed three times with PBS, fixed with a 4% paraformaldehyde solution for 15 minutes, and stained with 10 μM 1× Hoechst 33,342. The cells were imaged using a confocal laser scanning microscope (ZEISS, LSM 980).

**Biocompatibility Assay**

To evaluate the cytotoxic effects of nanoparticles and nanodroplets, cell viability was assessed using a CCK‑8 kit. In this experiment, bEnd.3 and U87‑MG cells were co‑incubated with various concentrations of nanocarriers for 24 hours, respectively. Subsequently, 10 µL of the CCK‑8 reagent was added and the cells were incubated in the dark for 1 hour. The cell absorbance was then measured using a microplate reader, and the background absorbance was subtracted from the measured values to obtain normalized absorbance readings. And the cell viability was calculated as follows:

*Cell viability (%) =*$\frac{{OD}_{mean value of the test group}-{OD}_{blank sample}}{{OD}_{mean value of the control group}-{OD}_{blank sample}}\times100$

**Blood-brain barrier *in vitro* model**

The bEnd.3 cells (2 × 10^4^/well) were seeded in the upper chamber of the transwell (TCS016024, BIOFIL), and C8-D1A cells (4 × 10^4^/well) were seeded in the lower chamber of the transwell. The upper chamber was filled with 200 μL of DMEM medium, and the lower chamber was filled with 800 μL of DMEM medium, incubated at 37°C in 5% CO_2_. The medium was replaced every other day. The successful construction of the cellular barrier was confirmed by measuring the transepithelial electrical resistance (TEER) value using a transepithelial resistance meter and the apparent permeability coefficient（P_app_）of FITC-dextran (4 kDa). TEER formula‌: TEER(Ω⋅cm^2^)=( R_sample_ - R_blank_) × A. R_sample_: Resistance of the cell monolayer (Ω); R_blank_: Resistance of the cell-free insert (Ω); A: Effective surface area of the membrane (cm²). P_app_ formula‌: P_app_ (cm/s) = (ΔQ/Δt) × V/ (A × C_0_). ΔQ/Δt: Rate of solute transport across the barrier (μg/s or mol/s); V: Volume of the receiver chamber (cm³); A: Effective diffusion area of the membrane (cm²); C_0_: Initial concentration in the donor chamber (μg/cm³ or mol/cm³).

***In vitro* BBB model transcytosis**

An integrated experimental protocol was conducted to evaluate the blood-brain barrier (BBB) permeability and siRNA delivery efficiency. Initially, the apical chamber medium was replaced with fresh medium supplemented with 10 μL of free Cy5‑siRNA, siRNA@NP, or siRNA@NP@ND to achieve a final siRNA concentration of 100 nM. Subsequently, ultrasound coupling gel was applied to the basolateral side of the *in vitro* BBB model, and ultrasound stimulation was administered using a probe (UT1041, Dundex) positioned on the gel-coated surface to enhance barrier permeability. After incubating the Transwell system at 37°C for 3 hours, 100 μL of basolateral chamber medium was collected, and the fluorescence intensity of transported siRNA micelles was measured using a microplate reader (excitation wavelength = 645 nm, emission wavelength = 670 nm) to calculate the transport efficiency. Additionally, post-intervention, the BBB model was immunostained with ZO-1 and GFAP antibodies and visualized using a confocal laser scanning microscope (LSM 980, ZEISS) to assess tight junction integrity and astrocyte activation, respectively.

**Assessment of BBB integrity *in vivo***

All animal experimental procedures were approved by the Animal Ethics Committee of Peking University‌. Healthy mice (BALB/c strain, 6-8 weeks old) received siRNA@NP@ND complexes via tail vein injection at a dosage of 2 mg Cy5-siRNA equivalent per kilogram body weight‌. After cranial shaving, a 2 × 3 mm² craniotomy window was surgically created to expose the brain tissue‌.The L12-3V transducer (128 elements, ATL, USA) was driven at a center frequency of 8.9 MHz, coupled with a programmable Verasonics Vantage 256 system (Kirkland, USA) for customized imaging and stimulation sequences‌. The acoustic protocols were implemented according to the methodology described by Dong et al [3]. To assess the effects of MB dose, ultrasound parameters, and stimulation duration on the integrity of the BBB, the animals were sacrificed approximately 6 hours after the injection of Evans Blue (EB). The mice were deeply anesthetized with tribromoethanol and perfused with heparinized normal saline via the left ventricle until a colorless perfusate was obtained from the right atrium; subsequently, the brain was harvested. The brain was then sectioned into multiple coronal slices. First, a qualitative analysis was performed to examine the extent and volume of EB extravasation within the brain as an indicator of BBB disruption. Second, a quantitative analysis was conducted to measure the amount of EB extravasation in the ultrasound-treated region. The brain tissue samples were weighed and placed in a 50% trichloroacetic acid solution. After homogenization, the mixture was centrifuged at 12,000 rpm for 20 minutes. The supernatant was diluted with anhydrous ethanol at a ratio of 1:3. Using a fluorescence spectrophotometer, the fluorescence intensity was measured at 620 nm. The EB extravasation in each brain tissue sample was calculated by linear regression from a standard curve generated using a series of diluted EB standard solutions and was expressed as the EB content per gram of brain tissue.

**Pharmacokinetics study**

BALB/c mice (female, 6–8 weeks old) and nude mice were purchased from Beijing Weitonglihua Company. A 200 μL PBS solution containing Cy5-siRNA@NP, Cy5-siRNA@NP@ND and naked siRNA was administered via tail vein injection into BALB/c mice (n = 3, at a dose of 2 mg Cy5-siRNA equiv./kg). Ultrasound stimulation was not administered in this experiment. At predetermined time points post-injection, approximately 50 μL of blood was collected from the orbital venous plexus of the nude mice. The collected blood samples were immediately mixed with 0.05 mL of lysis buffer (containing 1% Triton X‑100) and gently sonicated. Cy5‑siRNA was extracted by incubating the lysed blood samples with 0.5 mL DMSO at room temperature overnight, followed by centrifugation at 15,000 rpm for 30 minutes. The fluorescence intensity in the supernatant, corresponding to the Cy5 levels, was determined using a microplate reader (excitation wavelength = 645 nm, emission wavelength = 670 nm). The blood circulation followed a typical two‑compartment model, characterized by a rapid decline during the distribution phase and a prolonged elimination phase. Experimental data were fitted to a biphasic exponential decay model using Software Origin 9.1 to calculate the half‑lives of the two phases (t_1/2_,α and t_1/2_,β) according to the equation: y = A_1_ × exp(-x/t_1_) + A_2_ × exp(-x/t_2_) + y_0_, with t_1/2_,α = 0.693 × t_1_ and t_1/2_, β = 0.693 × t_2_.

***In vivo* biodistribution**

Mice in different dosing groups (2 mg Cy5‑siRNA equiv./kg) were imaged using the Lumina IVIS III system for whole‑body imaging (excitation wavelength = 649 nm, emission wavelength = 670 nm). Subsequently, the animals were euthanized via perfusion. The major organs (brain, heart, liver, kidney, spleen, and lung) were harvested, washed with cold PBS, and the Cy5 fluorescence in each organ was captured using the Lumina IVIS III system (excitation wavelength = 649 nm, emission wavelength = 649 nm). Furthermore, the collected brain tissues were rapidly frozen and sectioned into 5‑μm‑thick slices using a cryostat. The vasculature within the brain was then labeled with CD31, and the brain sections were counterstained with DAPI. All brain sections were imaged using a confocal laser scanning microscope (LSM 980, ZEISS).

**Histological Analysis**

After all treatments were completed, the following organs were collected from each group: brain, heart, liver, kidney, spleen, and lung. The collected organs were fixed in 4% formaldehyde for 24 hours, with the brain undergoing gradient sucrose dehydration. Subsequently, the organ samples were embedded in paraffin, sectioned into 5-μm-thick slices, mounted on slides, and stained with hematoxylin and eosin (H&E). The stained slides were examined using an optical microscope.

**Hematological Analysis**

BALB/c mice that received tail vein injections of either normal saline or siRNA@NP@ND were used. Twenty-four hours after injection, 1 mL of blood was collected via the ocular enucleation method. The blood samples were sent to Beijing Kang Jia Hong Yuan Biological Technology Co., LTD for routine hematological and biochemical analyses. Additionally, serum was separated from the blood by centrifugation at 3000 rpm for 10 minutes at 4°C and immediately stored at –20°C for further testing. The secretion levels of IL-6 and TNF-α in the serum were determined using a commercially available ELISA kit (Thermo Scientific, USA) in accordance with the manufacturer’s instructions.

**Statistical Analysis**

Quantitative data were analyzed using SPSS 27.0 software. All experimental results are presented as the mean ± standard deviation (SD) for each group. A two-tailed Student's t-test was used for comparisons between two groups, while comparisons among three or more groups were conducted using one-way analysis of variance (ANOVA) followed by Tukey’s post hoc test. A p-value of ≤0.05 was considered statistically significant, defined as follows: *p < 0.05, **p < 0.01, and ***p < 0.001.

Reference

[1] Montaldo, G., Tanter, M., Bercoff, J., Benech, N., & Fink, M. (2009). Coherent plane-wave compounding for very high frame rate ultrasonography and transient elastography. IEEE Transactions on Ultrasonics, Ferroelectrics, and Frequency Control, 56(3), 489-506.

[2] Melich, R., Bussat, P., Morici, L., Vivien, A., Gaud, E., Bettinger, T., & Cherkaoui, S. (2020). Development of a novel ultrasound contrast agent for targeted drug delivery. International Journal of Pharmaceutics, 587, 119651. https://doi.org/10.1016/j.ijpharm.2020.119651

[3] Dong, F., et al. (2024). Programmable ultrasound imaging guided theranostic nanodroplet destruction for precision therapy of breast cancer. Ultrasonics Sonochemistry, 105, 106854.


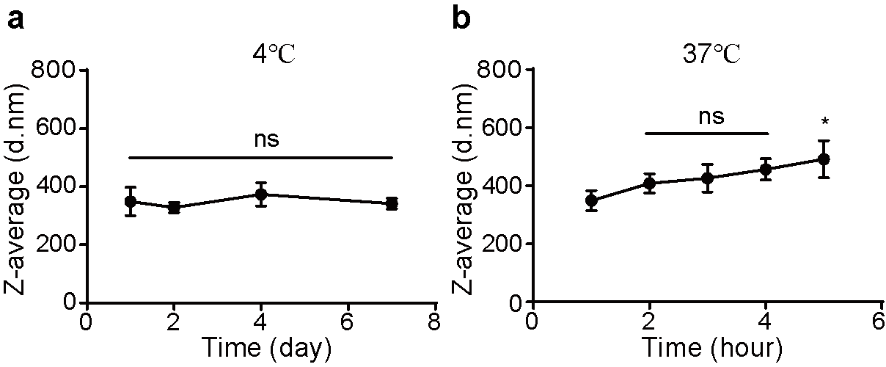


Figure S1. Size stability of siRNA@NP@ND stored at 4 °C and 37 °C (n=3). Data are presented as mean ± SD. Statistical analysis was performed using one-way ANOVA with Tukey’s post hoc test for multiple comparisons. *p < 0.05; ns: not significant.


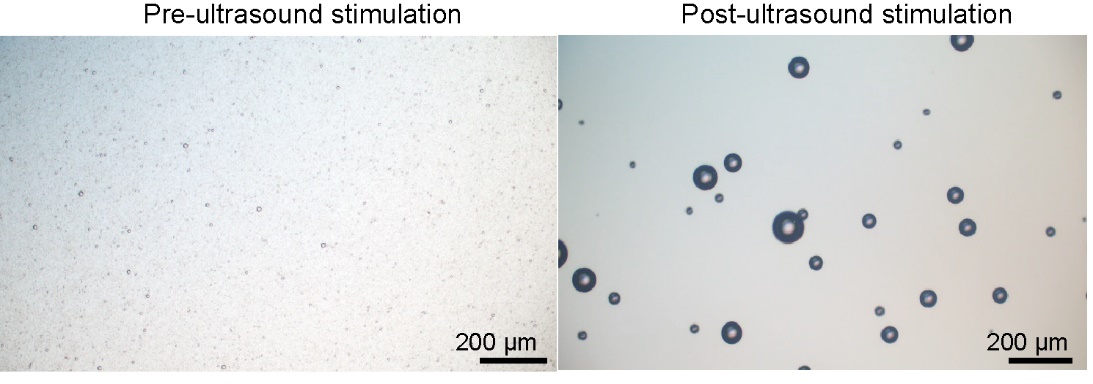


Figure S2. Representative bright-field microscopy images of siRNA@NP@ND before and after ultrasound exposure. Scale bar: 200 µm.


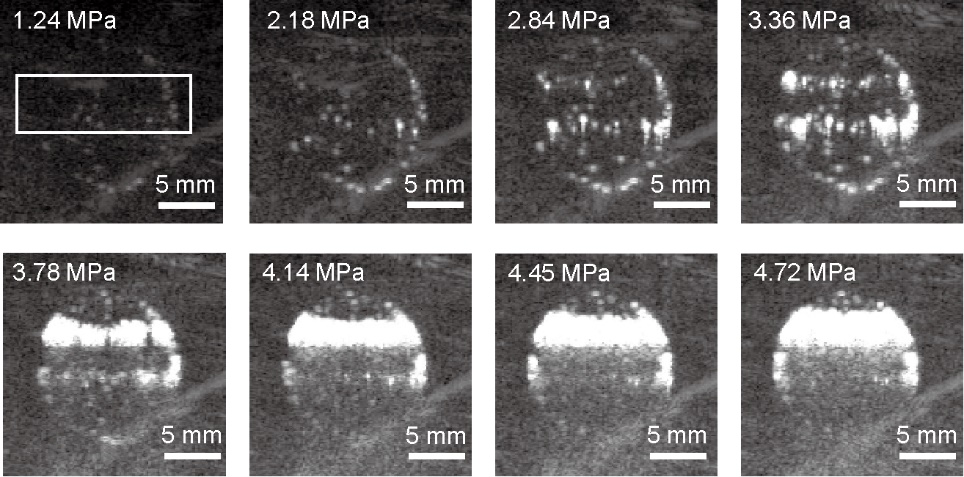


Figure S3. Agarose gel images of siRNA@NP@ND bursting under varying ultrasound intensities (1.24, 2.18, 2.84, 3.36, 3.78, 4.14, 4.45, and 4.72 MPa). White boxes outline the ultrasound-targeted regions. Scale bar: 5 mm.


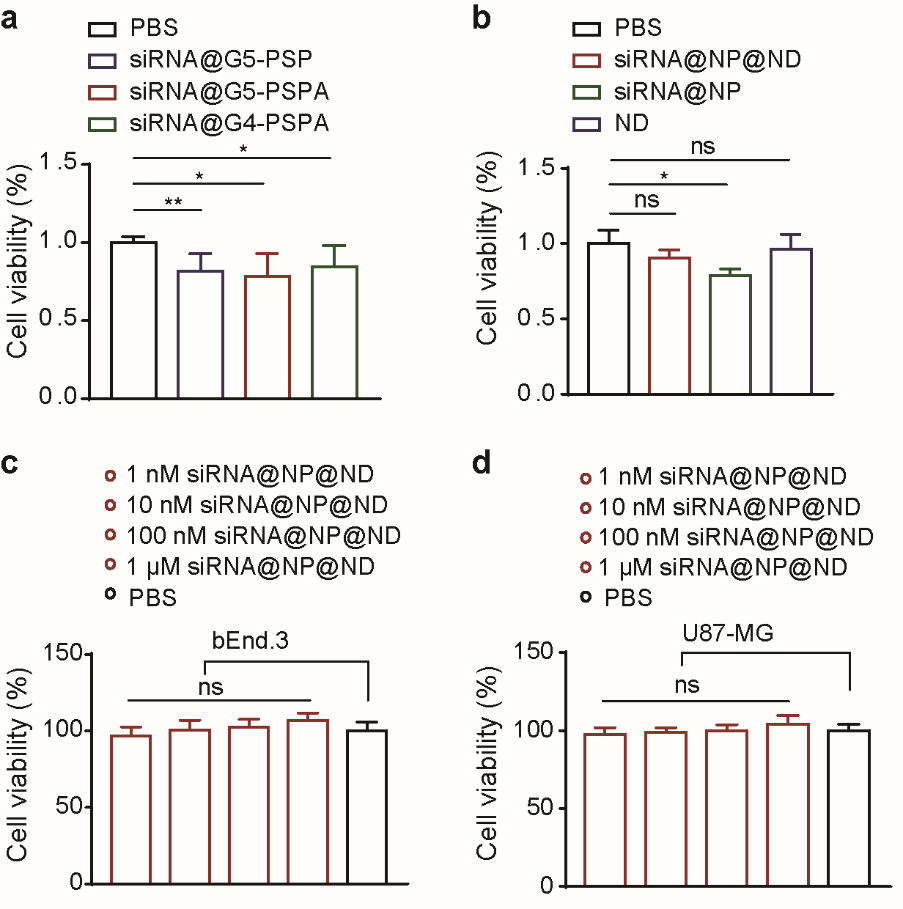


Figure S4. Cytotoxicity evaluation of diverse nanoparticles (a, b) and nanodroplets (c, d) (1 nM, 10 nM, 100 nM, 1 μM) on bEnd.3 endothelial cells and U87-MG cells (n=5). G5: fifth-generation PAMAM; G4: fourth-generation PAMAM; PSP: PAMAM-ss-PEG; PSPA: PAMAM-ss-PEG-Angiopep-2. Data are presented as mean ± SD. Statistical significance (*p < 0.05, **p < 0.01, ns: not significant) was determined by one-way ANOVA.


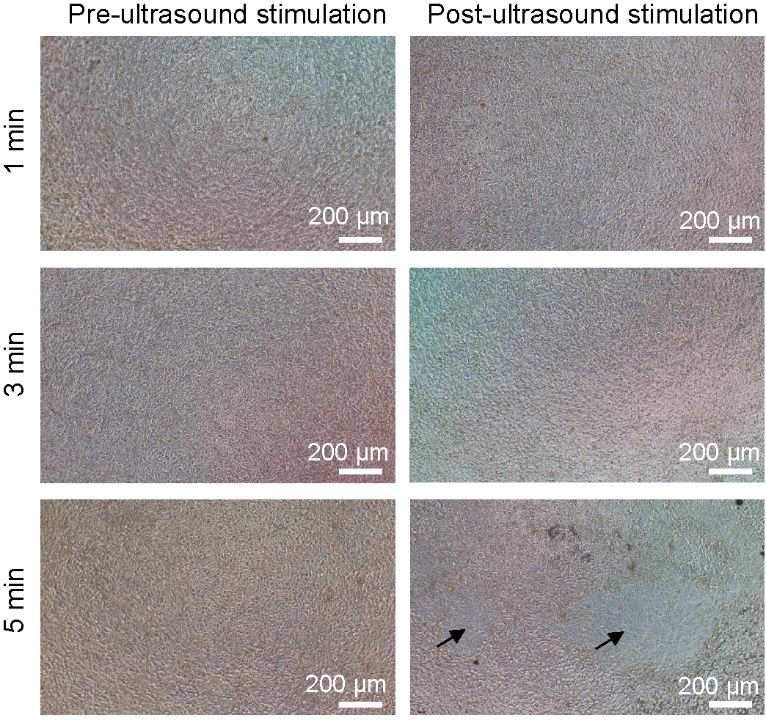


Figure S5. Bright-field microscopy images of the *in vitro* BBB model before and after ultrasound intervention at different durations (1 min, 3 min, 5 min). Barrier detachment (indicated by black arrows) was observed at 5 min. Scale bar: 200 μm.


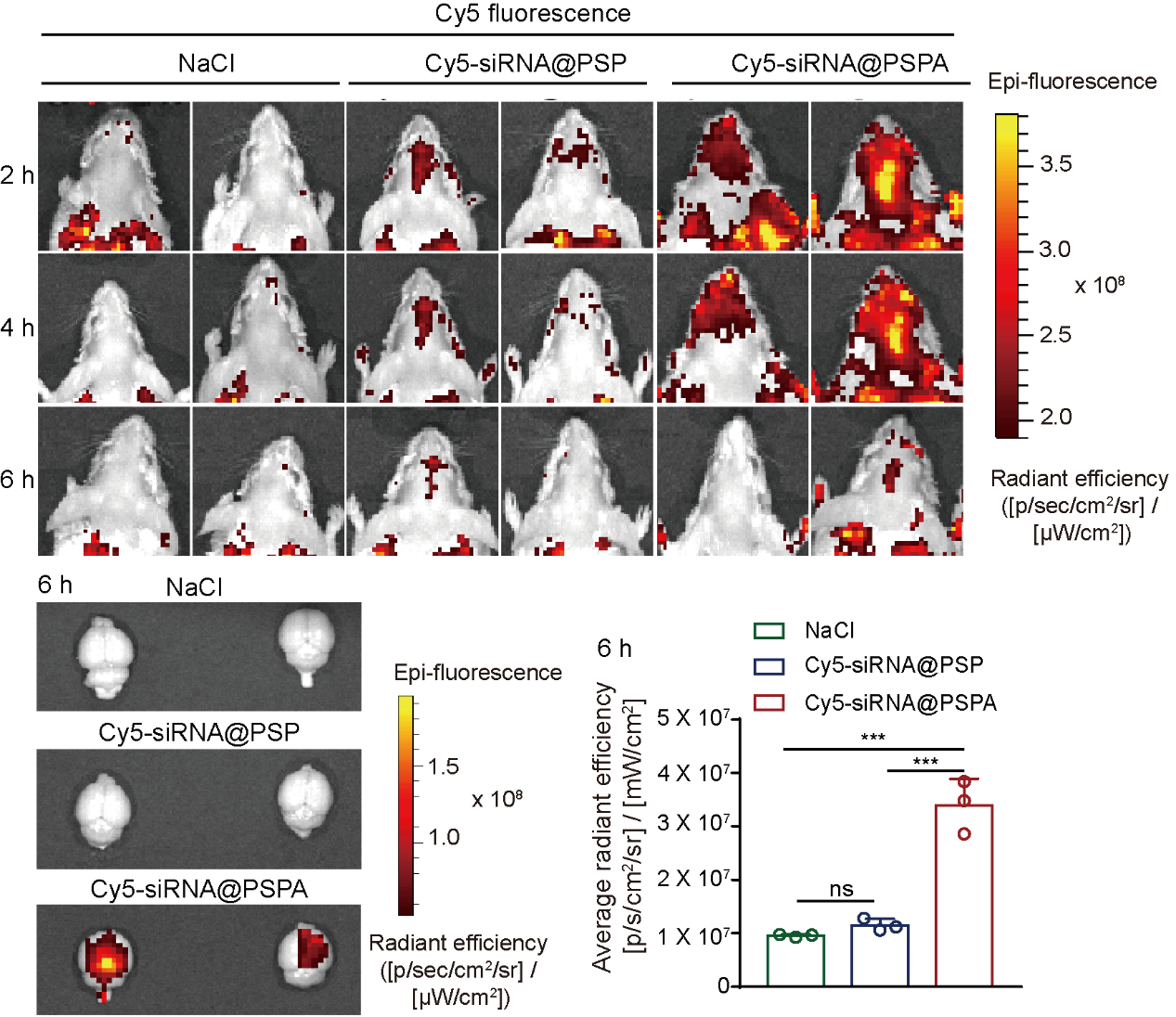


Figure S6. Time-dependent brain imaging and quantitative analysis of siRNA delivery. Top: *In vivo* brain imaging of Cy5-siRNA (2 mg/kg dose) at 2 h, 4 h, and 6 h post-administration in mice treated with NaCl, siRNA@PSP, or siRNA@PSPA (n=2 per group). Red fluorescence indicates Cy5-siRNA. Bottom: Brain imaging and quantitative analysis at 6 h post-administration (n=2 imaging, n=3 quantification). Data are presented as mean ± SD. Statistical significance (***p < 0.001, ns: not significant) was determined by one-way ANOVA.


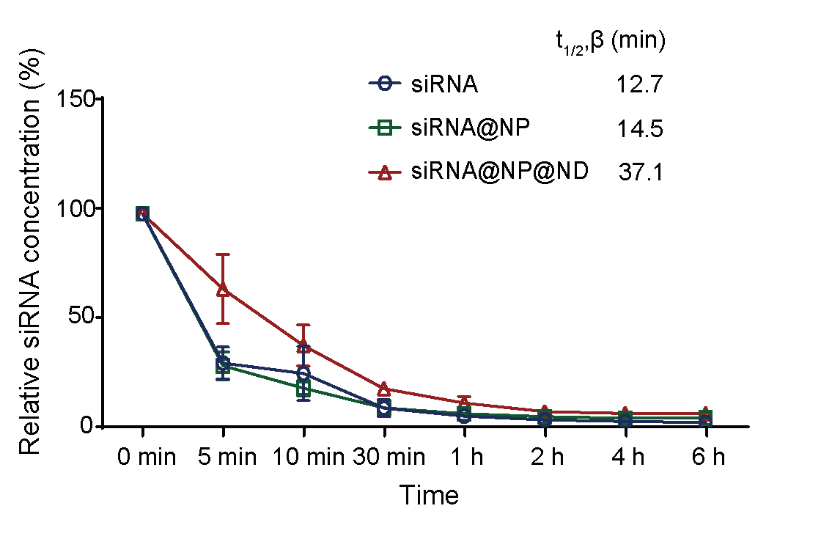


Figure S7. Blood concentration of siRNA over time (0 min, 5 min, 10 min, 30 min, 1 h, 2 h, 4 h, 6 h) in mice treated with free siRNA, siRNA@NP, or siRNA@NP@ND (n=3).


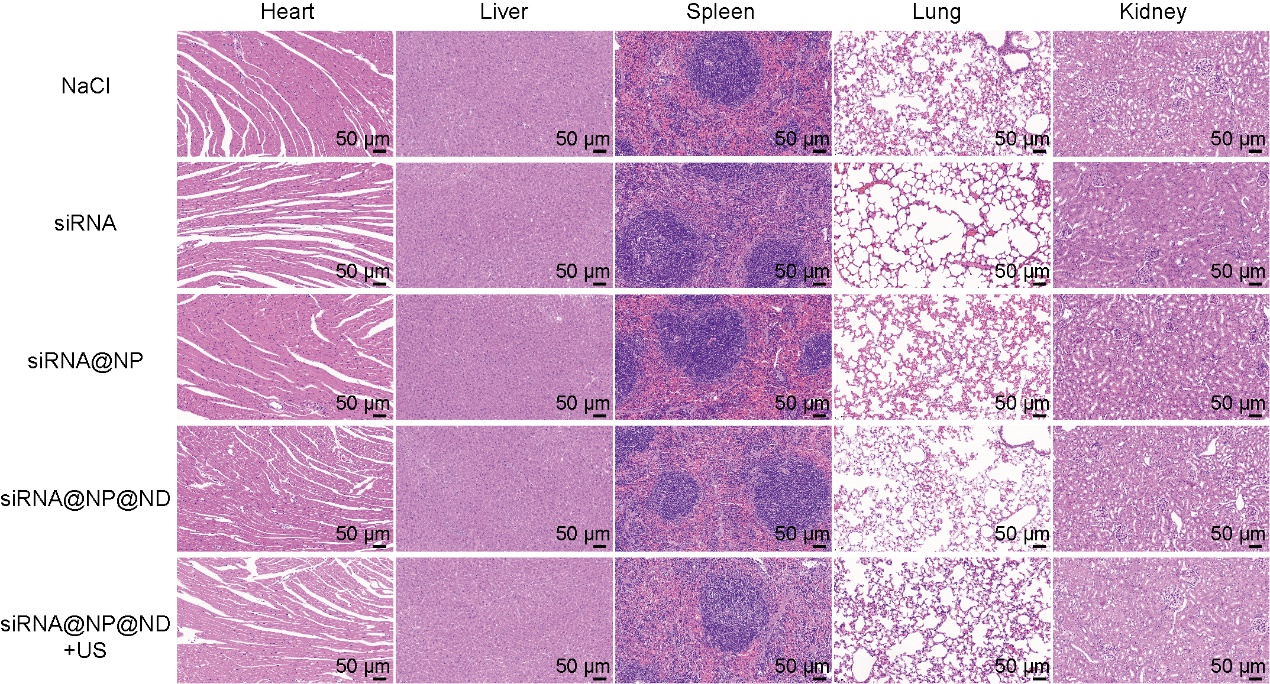


Figure S8. Hematoxylin and eosin (H&E) staining of brain, heart, lungs, liver, spleen, and kidneys. Scale bar: 50 μm.


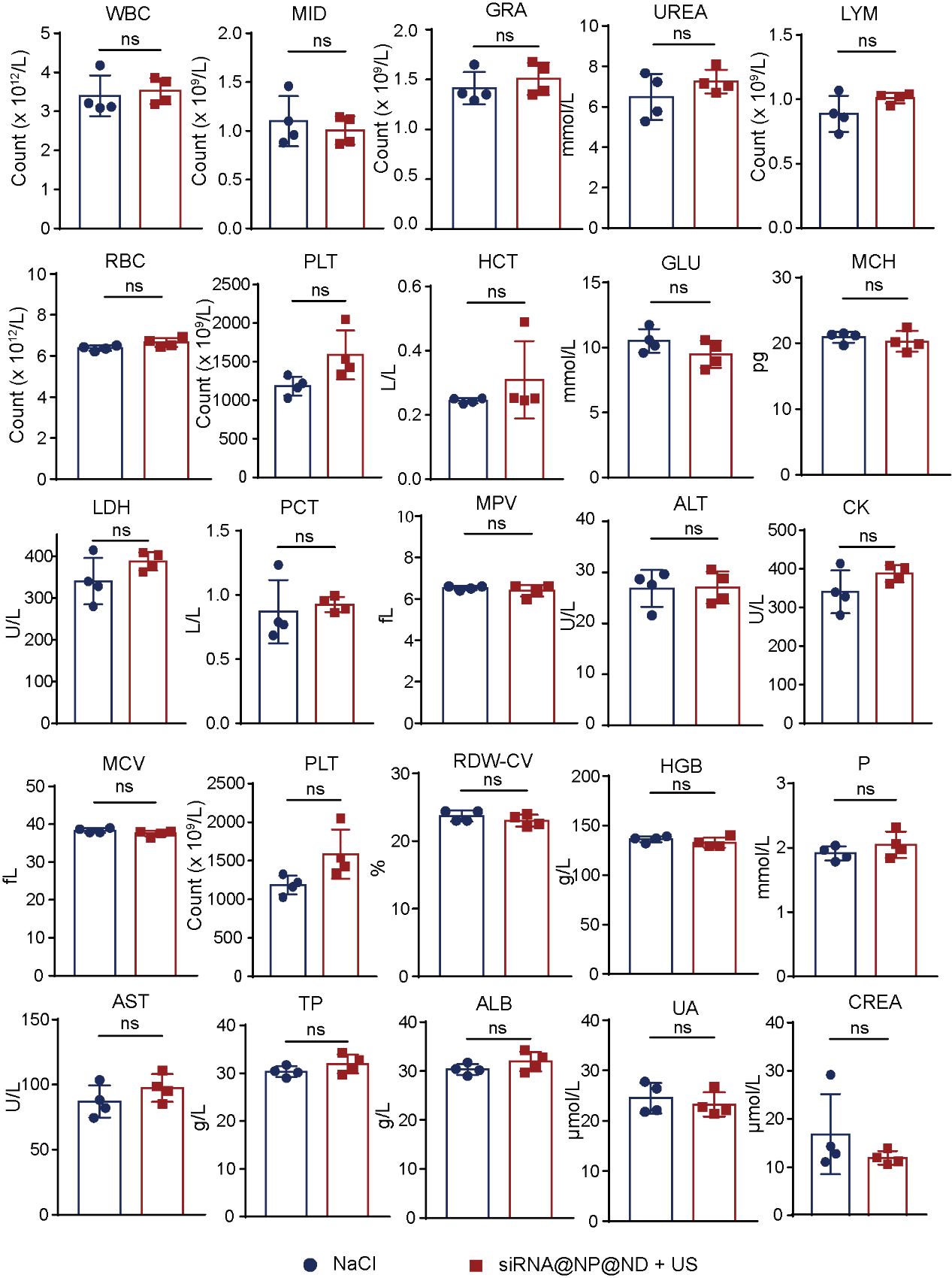


Figure S9. Hematological parameters in mice treated with NaCl or US + siRNA@NP@ND (n=4). Data are presented as mean ± SD. Statistical comparisons were performed using Student’s t-test. ns: not significant.
